# Adaptive Benefits of Antioxidant and Hormone Fluctuations in *Wedelia trilobata* Under Simulated Salt Stress with Nutrient Conditions

**DOI:** 10.3390/plants14030303

**Published:** 2025-01-21

**Authors:** Hong Yang, Bin Li, Ping Huang, Bin Zhang, Adeel Abbas, Zhiwei Xu, Huilei Yin, Daolin Du

**Affiliations:** 1 Institute of Environment and Ecology, School of Environment and Safety Engineering, Jiangsu University, Zhenjiang 212013, China; 2 School of Emergency Management, Jiangsu University, Zhenjiang 212013, China; 3 Water Conservancy and Lake Bureau of Daye City, Huangshi 435100, China; 4 Jingjiang College, Jiangsu University, Zhenjiang 212013, China

**Keywords:** invasive plant, simulated salt stress, nutrient conditions, plant antioxidant system, hormone contents

## Abstract

Salinity is one of the most significant environmental factors limiting plant development and productivity. Invasive plants could quickly respond to environmental changes, thus successfully achieving invasion. However, there is limited research on the mechanism of salt responses in invasive plants under different nutritional conditions. This study evaluated and compared the impact of salinity stress and nutrient application on physiological responses in the invasive plant *Wedelia trilobata* and native plant *Wedelia chinensis*. Mild salinity stress disrupted the growth of these two plants, significantly reducing their leaf and stem node number under a low nutrient condition. *W. trilobata* showed notable decreases in height and leaf number with high salinity stress regardless of nutrient levels, whereas it was observed only in the low nutrient state in *W. chinensis*. The negative effects of high salinity on both species were most evident in nutrient-poor environments. Under low salinity and nutrient stress, *W. trilobata*’s leaves exhibited increased levels of proline, MDA, CAT, and ABA, with decreased GA and IAA content. A low-salt environment favored *W. trilobata*’s competitive advantage, and nutrient enrichment appeared to enhance its invasive potential, in which process the plant antioxidant system and endogenous hormones contribute greatly. This study provides a theoretical foundation for predicting suitable growth areas for *W. trilobata* referring to the salt condition, guiding future strategies for preventing and controlling its invasive spread.

## 1. Introduction

Salinity has increasingly come under scientific scrutiny as a critical factor impeding the normal growth of plants, with approximately 8.7% of the world’s land being endangered by salinization, and China, in particular, experiencing a disproportionately severe soil salinization issue, constituting one-eighth of the global total [1,2]. Elevated salt concentrations under stressful conditions often lead to physiological disruptions in most plants, including impediments in different physiological activities [3]. These adverse effects may culminate in outcomes such as leaf abscission, reduced biomass, and, in extreme cases, plant mortality [4]. Salt stress raises intracellular osmotic pressure and leads to sodium ion toxicity, necessitating plant adaptations including ion balance regulation, phytohormone signaling, and antioxidant enzyme control [5]. The global proliferation of invasive plant species into new habitats underscores a pressing environmental challenge, as these invaders swiftly adapt to unfamiliar conditions, thereby enhancing their invasive potential through intricate interactions with a range of biotic and abiotic factors [6,7]. Many invasive plants show rapid adaptability to environmental stress [8,9]. Understanding the mechanisms behind invasive plant success is a primary focus in invasion ecology [10]. Invasive weeds consequently find themselves vulnerable to physiological disorders, a marked reduction in photosynthetic rates, and compromised growth [11]. Conversely, in nutrient-rich environments, invasive weeds gain a competitive edge over their native counterparts, flourishing amidst the challenges of saline ecosystems [12].

In the realm of invasive weeds, the paramount influence of salinity and nutrient application takes center stage, orchestrating a sophisticated interplay of enzymatic activities and endogenous hormones within plants [13]. Particularly in the challenging terrain of saline environments, salinity emerges as a formidable adversary, disrupting the operation of vital enzymes engaged in metabolic processes crucial for the very foundation of plant growth and development [14]. In this hostile setting, the disruption of enzymatic activities by salinity poses a substantial hurdle, affecting essential pathways such as photosynthesis and respiration, and thereby challenging the overall health and productivity of the plant [15,16]. Moreover, nutrient availability takes on the role of a linchpin, driving the efficiency of enzymatic reactions and wielding influence over endogenous hormones [17,18]. Specific nutrients serve as indispensable cofactors and substrates, forming the fundamental basis for achieving optimal enzymatic performance and regulating the delicate balance of hormones [19]. This nuanced interplay underlines the significance of precise nutrient provision and management, pivotal to ensuring robust enzymatic activities and hormone equilibrium, ultimately enhancing the resilience and performance of plants in various environmental contexts [20]. Endogenous hormones, the masterminds behind plant growth and development, are finely attuned to the persuasive influences of salinity and nutrient availability [21,22]. They govern the intricate orchestration of hormones, including auxins, gibberellins, and abscisic acid, through their synthesis and distribution [23]. In saline conditions, the closure of stomata is prompted to conserve water, while nutrient-rich environments encourage the activation of hormone pathways that expedite growth and mold the plant’s form and function [24]. This intricate interplay, entailing salinity, nutrient application, enzymatic activities, and endogenous hormones, carries profound significance within the realm of agriculture [25].

The strategies developed to oversee and alleviate the effects of salinity on enzymatic processes and hormone regulation are undeniably crucial, particularly in regions plagued by the enduring challenge of soil salinity [26]. The precise delivery of nutrients and skilled management of salinity levels emerge as pivotal tools to enhance plant resilience. *W. trilobata*, native to tropical America, is a highly invasive species found in southern China, notably in the Guangxi and Hainan provinces [27,28]. It is listed among the “World’s 100 most malignant invasive species” by the IUCN [29]. Many studies have explored diverse facets of *W. trilobata* in the context of abiotic stresses, yet its adaptation to salt stress under varying nutrient conditions remains notably unexamined and unreported. In this study, we used *W. chinensis*, a closely related Asteraceae plant in China, as the native control plant [28], and compared how simulated salt stress affects the growth, antioxidant enzyme activities, and endogenous hormone ratios of *W. trilobata* and *W. chinensis* under varying nutrient conditions. Our specific objectives were to answer the following questions: (1) Might *W. trilobata* outcompete native species under simulated salt stress with various nutrient conditions? (2) In this process, what are the differences in antioxidant systems and plant hormone responses between these two species? The findings will offer insights into predicting suitable habitats for *W. trilobata* and inform strategies for future invasion prevention and control.

## 2. Materials and Methods

### 2.1. Experimental Materials

The invasive *W. trilobata* was collected from the urban precincts of Haikou city, Hainan province (19°31′~20°04′ N, 110°07′~110°42′ E). Concurrently, *W. chinensis* was gathered from the greenhouse of School of Environment and Safety Engineering at Jiangsu University. The stem segments of *W. trilobata* and *W. chinensis*, exuding remarkable vigor and steadfast uniformity in both length and girth, were handpicked to ensure the retention of two nodes for each stem segment. These selected cuttings were cultured in round plastic pots, measuring 90 millimeters (mm) in diameter, 60 mm in width, and 80 mm in height as botanical specimens. As the experiment’s foundation, the river sand was measured with exactitude, allotting 360 g for each individual pot. The chosen stem segments were arranged in a vertical posture within their respective pots, watered with deionized water, and housed within the greenhouse. After a span of 4 to 5 days, the nascent buds were ushered into the realm of emergence, marking the moment for subsequent treatments.

### 2.2. Experimental Method and Design

The bifactorial design in this experiment was as follows: factor 1—three different salt stress levels: no salt (0 mM), low salt (100 mM, equivalent to a salt content of 0.117%), and high salt (200 mM, corresponding to a salt content of 0.234%); and factor 2—three divergent nutrient levels: low nutrient level (0.1 × Hogland standard nutrient solution to replicate circumstances of nutrient deprivation), normal nutrient level (0.5 × Hogland standard nutrient solution to mimic the requisites for typical plant growth), and high nutrient level (1.0 × Hogland standard nutrient solution to imitate a nutrient-rich milieu). For each experimental treatment, there were 5 replicates and 2 plant species, resulting in a total of 90 pots. Three plants of each experimental treatment replicates were randomly selected for data analysis. Simultaneously, in order to mitigate the salinity-induced repercussions stemming from the 200 mM salt treatment, incremental escalations from 100 mM NaCl were implemented at intervals of 24 h, with a concomitant addition of 100 mL on each occasion to reach the desired concentration. Following the salt stress intervention, the aforementioned triad of nutrient levels was applied every alternate day, with each plant receiving a 100 mL aliquot of water.

## 3. Data Collection

### 3.1. Phenotypic Growth Indicators

After a growth period of 30 days post-cultivation, the plants grown in each receptacle were carefully harvested, and relevant parameters underwent assessment. The evaluation encompassed a range of growth indices, including plant height, leaf number, stem node number, and root length. In the interest of precision, each index underwent triplicate measurements, and the resultant data were subsequently averaged for statistical rigor. The enumeration of leaf and stem nodes was carried out manually, while plant height and root length were quantified using Image J 1.38e (https://imagej.nih.gov/ij/) (accessed on 15 October 2021), which was employed after capturing digital images with a specialized camera apparatus. The freshly harvested leaves, stems, and roots were thoroughly cleansed with pristine water, briefly exposed to a temperature of 105 °C for a duration of 10 min, and thereafter transferred to a drying oven, where they underwent desiccation at 65 °C for a span of 72 h. The determination of leaf dry weight, stem dry weight, and root dry weight was carried out with utmost precision, employing an analytical balance of exactitude. The aggregate of these individual measurements constituted the overall plant biomass, whereas the ratio of root dry weight to aerial dry weight delineated the root–shoot ratio.

### 3.2. Biochemical Indicators

Measurements of the antioxidant system include the determination of proline, malondialdehyde (MDA), peroxidase (POD), and catalase (CAT) content. Where proline was measured by the acid ninhydrin method [30], MDA was determined using the thiobarbituric acid method [31], POD activity was determined by the guaiacol chromogenic method and CAT activity was measured by the UV absorption method [32].

### 3.3. Endogenous Hormones

The content of gibberellin (GA), auxin (IAA), and abscisic acid (ABA) in *W. trilobata* and *W. chinensis* was determined using an enzyme-linked immunosorbent assay (ELISA) kit [33]. The endogenous hormone kit was purchased from the Crop Chemical Control Laboratory, China Agricultural University (Beijing, China).

## 4. Data Analysis

The analytical software SPSS (version 22.0) was duly employed to carry out both univariate and multivariate analyses of variance (ANOVA) as well as correlation analyses for each individual factor under consideration. The two-way analysis of variance of the two factors was shown in Table 1 and Table 2, and the Pearson correlation analysis was shown in Table 3 and Table 4. Concurrently, the software Origin (version 8.1) was harnessed to graphically represent the outcomes derived from the rigorous statistical examination of the dataset. In the pursuit of comprehensive insights, multiple comparisons were meticulously executed through the application of the Student–Newman–Keuls test, facilitating a detailed exploration of the observed disparities. It is noteworthy that, throughout this intricate investigative process, a threshold of statistical significance was meticulously set at a value of *p* ≤ 0.05 for all the biomarkers, thereby ensuring that only the most robust and substantiated findings were considered noteworthy and, by extension, actionable.

## 5. Results and Analysis

### 5.1. Effects of Different Salinity Level on the Growth of Wedelia trilobata and W. chinensis Under Different Nutrient Conditions

**Figure 1:**
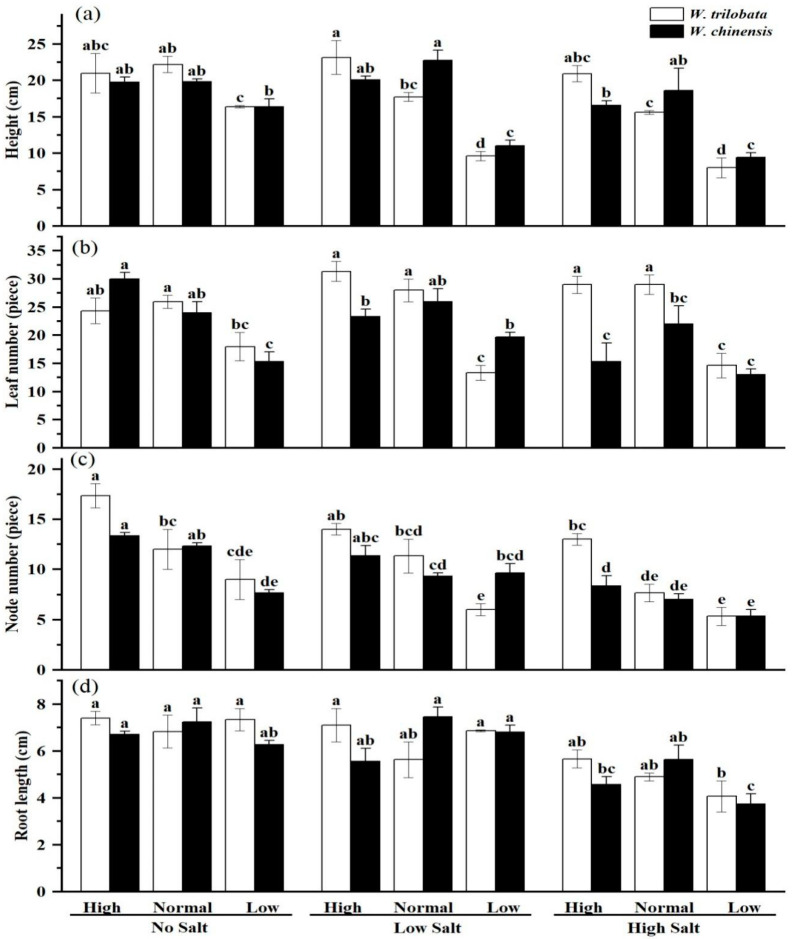
Effects of simulated salt stress on plant height (**a**), leaf number (**b**), nude number (**c**), and root length (**d**) of two plants under different nutrient conditions. The data are presented as the mean ± SE (n = 3), and sharing the different letters indicates significant differences between treatments (*p* < 0.05).

**Figure 2:**
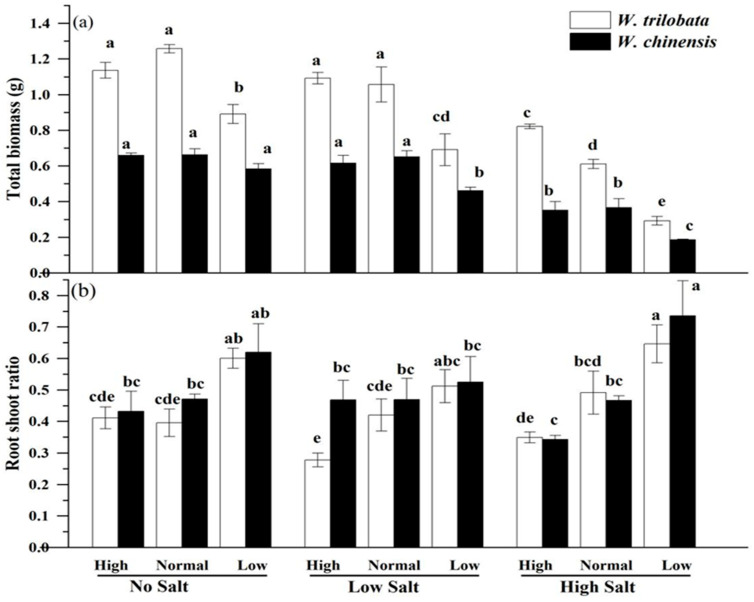
The impact of simulated salt stress on the total biomass (**a**) and root–shoot ratio (**b**) of two plants under different nutrient conditions. The data are presented as the mean ± SE (n = 3), and sharing the different letters indicates significant differences between treatments (*p* < 0.05).

In the absence of salinity stress, incremental or decremental alterations in nutritional levels exhibited no discernible influence on the growth parameters of *W. trilobata* in comparison to standard nutrient conditions (Figure 1a–d). However, for *W. chinensis*, the decrease in leaf number and stem nodes was notably significant under low nutrient conditions (Figure 1b,c). Under conditions of low salinity stress, the plant height and leaf number of both *W. trilobata* and *W. chinensis* exhibited substantial reductions to varying degrees in response to low nutrient levels (Figure 1a,b). When subjected to high salinity stress, low nutrient levels manifested distinct impacts on plant height, as contrasted with standard nutrient conditions (Figure 1a). Furthermore, low nutrient levels had a noticeable effect on the leaf number of *W. trilobata* (as exemplified in Figure 1b). However, for *W. chinensis*, the pronounced influence of high salinity stress on plant height, leaf number, and root length was only observed under conditions of low nutrient availability (Figure 1a,b,d). In the absence of salinity stress, low nutrient levels demonstrated a significant effect on the total biomass and root–shoot ratio of *W. trilobata* in comparison to standard nutrient conditions (Figure 2a,b; *p* < 0.05), resulting in a 21.6% decrease in the total biomass. Conversely, *W. chinensis* appeared unaffected (Figure 2a). Under both high and low salinity stress, low nutrient levels led to a substantial reduction in the total biomass of *W. trilobata* compared to standard nutrient conditions (Figure 2a; *p* < 0.05). Conversely, the total biomass of *W. chinensis* only declined under low nutrient conditions in the presence of high salinity stress (Figure 2a; *p* < 0.05). Irrespective of high salinity stress conditions, Low nutrition reduced the root–shoot ratio of both plant species when contrasted with standard nutrient conditions (Figure 2b). Correlation analysis results indicated a positive association between the total biomass of *W. trilobata* and all corresponding growth indicators. The root–shoot ratio exhibited a notably negative correlation with plant height, leaf number, and stem node number (as presented in Table 4; *p* < 0.01). In the case of *W. chinensis*, the total biomass was significantly positively correlated with all the pertinent growth indicators, while the root–shoot ratio displayed a significant negative association with plant height and leaf number (Table 4; *p* < 0.05).

### 5.2. Effects of Different Salinity Levels on the Antioxidant Enzyme Activities of Wedelia trilobata and W. chinensis Under Different Nutrient Conditions

**Figure 3:**
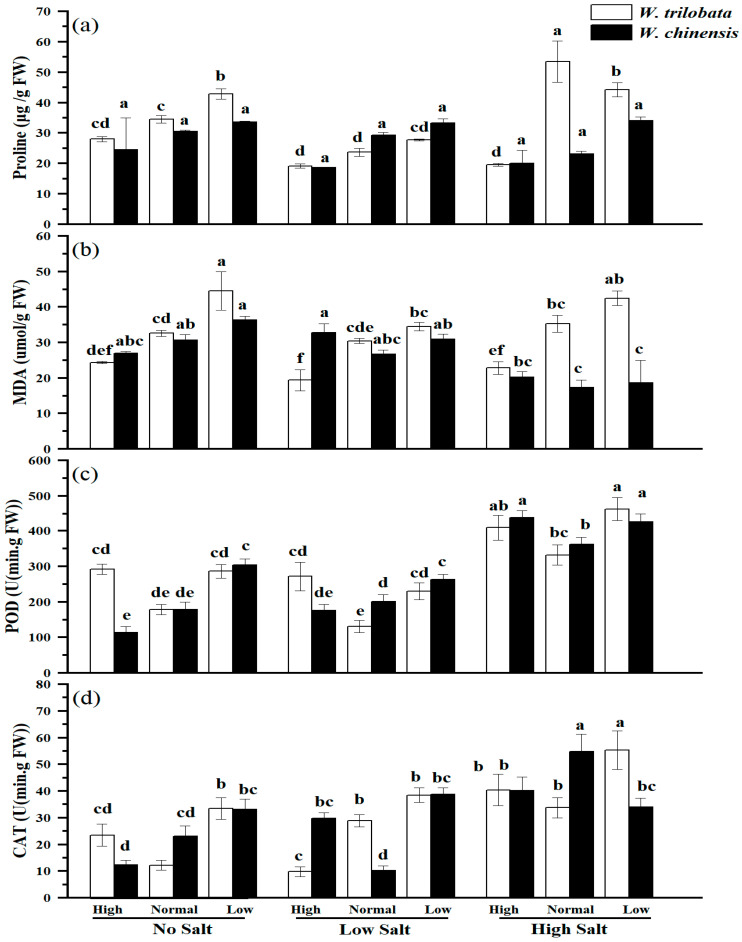
The impacts of simulated salt stress on proline (**a**), malondialdehyde (**b**), peroxidase (**c**), and catalase (**d**) of two plants under different nutrient conditions. The data are presented as the mean ± SE (n = 3), and sharing the different letters indicates significant differences between treatments (*p* < 0.05).

As depicted in Figure 3, in the absence of salt-induced stress, the concentrations of proline, MDA, and CAT in *W. trilobata* exhibited a notable increase under conditions of diminished nutrient availability, in stark contrast to standard nutrient levels, while the levels of POD did not change significantly and remained unaltered (Figure 3a–d). Within the same treatment framework, solely the POD content displayed a substantial increment (Figure 3c), while the remaining antioxidant indices exhibited no significant deviations for *W. chinensis*. Under low salt stress, heightened nutrient levels resulted in a significant reduction in MDA content in *W. trilobata* compared to the standard nutrient levels, while *W. chinensis* remained relatively unchanged. Notably, both elevated and reduced nutrient levels elicited an augmentation in the POD content for *W. trilobata*, with this effect being solely observable at diminished nutrient levels for *W. chinensis* (Figure 3c). In the context of low salt stress, the nutritional status impacted the CAT content of the two species disparately, giving rise to a substantial decline in CAT content for *W. trilobata* under heightened nutrient levels, while *W. chinensis* experienced a marked increase in CAT content, irrespective of whether nutrient levels were elevated or reduced (Figure 3d, *p* < 0.05). Under high salt stress conditions, heightened nutrient levels engendered a pronounced reduce in proline and MDA concentrations for *W. trilobata*, whereas these fluctuations did not significantly influence *W. chinensis*. For both *W. trilobata* and *W. chinensis*, the levels of POD and CAT underwent substantial modifications in response to diminished nutrient availability, with POD content experiencing a notable increment under low nutrient levels for *W. trilobata*, while for the same species, CAT content exhibited a significant reduction under reduced nutrient conditions (Figure 3). The outcomes of the correlation analysis unveiled that proline content in *W. trilobata* exhibited a notable and adverse relationship with plant height, node count, total biomass, and the root–shoot ratio, while the MDA content revealed a remarkably strong inverse correlation with plant height, leaf number, stem count, total biomass, and the root–shoot ratio. Furthermore, the POD content demonstrated a significant and negative association with root length and total biomass, whereas the CAT content exhibited a substantial and negative relationship with all the growth indicators (Table 3, *p* < 0.05). Conversely, the proline content in *W. chinensis* exhibited a significant association with plant height and the root–shoot ratio. The MDA content displayed a noteworthy positive correlation with the stem node count and total biomass. Intriguingly, the POD content exhibited an extremely strong and inverse relationship with all the growth indicators, while the CAT content showcased a significant and negative association with the stem node count, root length, and total biomass (Table 4, *p* < 0.05).

### 5.3. Effects of Simulated Salinity Stress on Endogenous Hormone Ratio of Wedelia trilobata Under Different Nutrient Conditions

**Figure 4:**
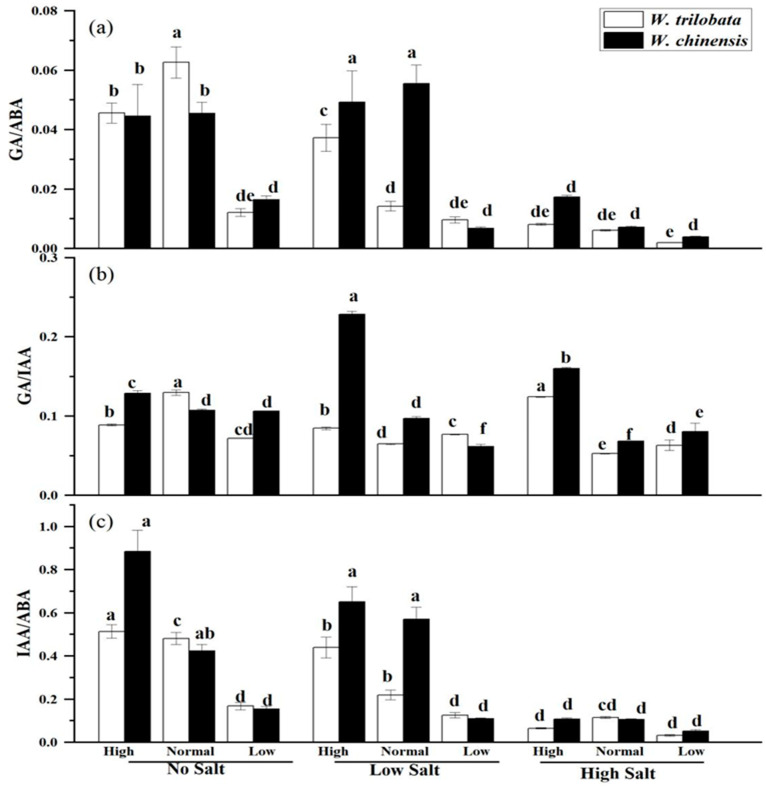
Influence of simulated salt stress on the ratios of GA to ABA (**a**), GA to IAA (**b**), and IAA to ABA (**c**) of two plants under different nutrient conditions. The data are presented as the mean ± SE (n = 3), and sharing the different letters indicates significant differences between treatments (*p* < 0.05).

As shown in Figure 4, in the absence of salt stress, high or low nutrient levels significantly reduced the GA/ABA and GA/IAA values of *W. trilobata* compared to normal nutrient levels (Figure 4a,b, *p* < 0.05), while the IAA/ABA values increased at high nutrient levels and were significantly reduced at low nutrient levels (Figure 4c, *p* < 0.05). In the absence of salt stress, low nutrient levels significantly reduced the GA/ABA and IAA/ABA values for *W. trilobata* (Figure 4a,c, *p* < 0.05). At low salt stress, high nutrient levels significantly increased the GA/ABA and GA/IAA values, while low nutrient levels significantly reduced the GA/ABA and IAA/ABA values of the *W. trilobata* (Figure 4a,c, *p* < 0.05). Low nutrient levels significantly reduced the ratio of three endogenous hormones for *W. chinensis* (Figure 4a–c, *p* < 0.05). Under high salt stress, the GA/IAA values in the leaves of both plants increased significantly with high and low nutrient levels (Figure 4b, *p* < 0.05). The results of the correlation analysis showed that the GA/ABA values were significantly positively correlated with plant height, node number, root length, and total biomass for *W. trilobata* and negatively correlated with the root–shoot ratio, MDA, POD, and CAT content. The GA/IAA values showed a significant positive correlation with plant height, stem node number, and total biomass, and a significant positive correlation with the root–shoot ratio, proline, and MDA content. The IAA/ABA values showed significant positive correlations with plant height, stem node number, root length, and total biomass, and a significant negative correlation with MDA, POD, and CAT (Table 3, *p* < 0.05). For *W. chinensis*, the GA/ABA values were positively associated with plant height, stem node number, leaf number, and total biomass, while there was a significant negative correlation with proline, POD, and CAT content. The IAA/ABA values showed a significant positive correlation with all growth indicators and showed a significant negative correlation with POD and CAT content (Table 4, *p* < 0.05).

## 6. Discussion

Elevated salinity, as a potent environmental determinant, exerts a profound impact on the intricate orchestration of plant growth and developmental processes, precipitating adverse consequences and unsettling the established norms of vegetative expansion [34,35]. Concurrently, it is imperative to acknowledge that the nutritional context in which plants reside can wield a significant influence over the colonization and proliferation of invasive plant species [36]. Within the scope of this scientific inquiry, we have meticulously scrutinized the implications of simulated salt stress on the growth dynamics of two distinct plant species across a spectrum of nutritional scenarios. Our all-encompassing analysis, encompassing both morphological metrics and biomass evaluations, has unveiled compelling insights. Under salt stress, seed priming with SWE increased root length, shoot length, root dry weight, chlorophyll content, and antioxidant enzymes activity compared with non-treated plants [37]. Under no salt stress, nutritional variations primarily manifested as an increase in leaf number and stem nodes for *W. chinensis*, whereas for *W. trilobata*, these variances were chiefly evident in the aspects of total biomass and the root–shoot ratio [38]. In the presence of high salt stress, changes in nutrient levels had a significant impact on both plants; high and low nutrient levels inhibited plant height and total biomass in *W. trilobata*, while in *W. chinensis*, only plant height, leaf number, and root length significantly decreased under low nutrient conditions. This suggests that higher nutrient levels can partially mitigate the negative effects of salt stress on plants under low salt stress conditions [39]. Enhancing nutrient utilization across varying salinity levels, as demonstrated in our study, ameliorates the effects of abiotic stress in both plants, as evidenced in the case of the invasive plant *Alternanthera philoxeroides*, underscoring the potential of nutritional conditions to alleviate stress-induced inhibition [40]. In resource-rich habitats, lower root–shoot ratios signify heightened competitiveness, aligning with the expectation of optimized biomass allocation for efficient resource capture [41,42]. In a high nutrient environment, salt reduced the root–shoot ratio of *W. trilobata*, indicating a shift in plant priorities toward optimizing aboveground growth for enhanced photosynthesis and improved survival [43]. The augmentation of nitrogen and phosphorus, coupled with the utilization of various Hogland nutrient solutions, fosters the growth and competitive advantage of invasive weeds [44,45]. Our study highlights the impracticality of this premise under high-salinity conditions, potentially arising from the adverse impact of high salt stress on plant cells, leading to metabolic disruptions and the impairment of the plant’s antioxidant system [46]. In the absence of salt stress, *W. trilobata* showed increased proline, MDA, and CAT levels in low nutrient conditions, with no significant change in POD. Under low salt stress, both *W. trilobata* and *W. chinensis* had lower proline, MDA, POD, and CAT levels in high nutrient environments, while proline and MDA increased in low nutrient conditions [47]. In high salt stress, elevated nutrition levels significantly raised proline and MDA in *W. trilobata* but had no significant impact on *W. chinensis*. The changes in the antioxidant system indexes were not obvious, as only the content of POD increased significantly [48]. Overall, plants can efficiently mitigate the detrimental effects of excessive Reactive Oxygen Species (ROS) by harnessing the synergistic capabilities of POD and CAT [49]. Under low salt stress, both *W. trilobata* and *W. chinensis* had lower proline, MDA, POD, and CAT levels in high nutrient conditions, but higher proline and MDA in low nutrient conditions. However, under high salt stress, high nutrition significantly increased proline and MDA in *W. trilobata*, with no notable effect on *W. chinensis*. These results highlight the intricate interactions between salt stress, nutrient availability, and plant antioxidant system responses [50]. In response to high salt stress, *W. trilobata* elevates proline content to enhance cellular osmotic adjustment, thereby stabilizing plant proteins and mitigating the damage inflicted by salt ions on plant cells. The alteration in MDA content in plants further lends support to this hypothesis [51]. Under the influence of drought stress, the ABA levels in the leaves and roots of *W.trilobata* were significantly increased compared with the control group [52]. When we measured the endogenous plant hormone levels under different conditions, we noticed a marked reduction in the GA/ABA ratio in the leaves of both plants when the nutrient levels were altered, particularly in comparison to the normal nutrient level [53]. Low salt stress had the greatest impact on hormone ratios in *W. chinensis* with low nutrient levels, causing a decrease in all three hormone ratios in this specific condition [54]. High salt stress increased the GA/IAA ratio in both plant species under both high and low nutrient conditions. The GA/ABA and IAA/ABA ratios under salt stress were positively correlated with plant height and total biomass [55]. The hormone ratios in *W. trilobata* strongly correlate with growth parameters, highlighting the roles of GA, IAA, and ABA in salt adaptation. In changing environments, increasing ABA helps regulate stomatal closure during drought, reducing water loss and enhancing drought tolerance [56].

## 7. Conclusions

This study demonstrates that the impact of simulated salt stress on *Wedelia trilobata* and *W. chinensis* is significantly influenced by nutrient levels. Low nutrient levels exert a more pronounced effect on both plants compared to high nutrient levels, regardless of salt conditions. Higher salt concentrations exacerbate the negative effects on plant growth and development. Under low salt stress, *W. trilobata* adapts by increasing proline, MDA, and CAT content in leaves while reducing GA/IAA content, indicating that a low-salt environment is more favorable for its competitiveness. Additionally, nutrient enrichment appears to enhance *W. trilobata*’s invasive potential. In this process, plant antioxidant system and endogenous hormones contribute greatly. To control and prevent the spread of *W. trilobata*, the application of saline water solutions and artificial soil salt enrichment are recommended as effective measures. These strategies can inhibit the plant’s propagation and mitigate its invasive impact. Further study on the competition with multi-species, which are coexisting and competing with *Wedelia trilobata* in practice, will provide more support for our research findings. Overall, this study provides a theoretical foundation for predicting suitable growth areas for *W. trilobata* and offers valuable insights for guiding future efforts in invasion prevention and control.

## Figures and Tables

**Table 1:** Analysis of variance results of the effects of salt and nutrition on *Wedelia trilobata*.

*Wt* Parameters	Salt		Nutrition		Salt × Nutrition	
	F	*p*	F	*p*	F	*p*
**PH**	9.653	0.001	42.472	<0.001	3.427	0.03
**LN**	0.419	0.664	32.012	<0.001	1.882	0.157
**NN**	2.188	0.141	10.256	0.001	0.251	0.905
**RL**	15.22	<0.001	2.436	0.116	1.209	0.341
**TB**	76.457	<0.001	49.404	<0.001	2.871	0.053
**RSR**	3.289	0.061	21.522	<0.001	1.173	0.356
**Proline**	29.772	<0.001	37.071	<0.001	10.639	<0.001
**MDA**	5.597	0.013	45.933	<0.001	0.851	0.511
**POD**	41.974	<0.001	17.317	<0.001	0.821	0.529
**CAT**	20.634	<0.001	18.081	<0.001	3.917	0.019
GA/ABA	46.861	<0.001	36.61	<0.001	12.993	<0.001
GA/IAA	7.473	0.004	43.59	<0.001	0.219	0.924
IAA/ABA	48.753	<0.001	25.182	<0.001	12.206	<0.001

Notes: *p* < 0.05 indicates a significant difference, *p* > 0.05 means no significant difference.

**Table 2:** Analysis of variance results of the effects of salt and nutrition on Wedelia chinensis.

*Wc* Parameters	Salt		Nutrition		Salt × Nutrition	
	F	*p*	F	*p*	F	*p*
**PH**	7.339	0.005	33.814	<0.001	2.838	0.055
**LN**	4.41	0.028	6.304	0.008	1.675	0.199
**NN**	17.397	<0.001	10.691	0.001	2.132	0.119
**RL**	22.25	<0.001	7.416	0.004	1.879	0.158
**TB**	84.638	<0.001	17.916	<0.001	0.966	0.45
**RSR**	0.135	0.875	8.182	0.003	1.656	0.204
**Proline**	0.895	0.426	9.356	0.002	0.555	0.698
**MDA**	21.139	<0.001	1.558	0.238	1.731	0.187
**POD**	114.331	<0.001	20.27	<0.001	7.878	0.001
**CAT**	24.65	<0.001	3.56	0.05	13.604	<0.001
**GA/ABA**	9.234	0.002	3.158	0.067	1.23	0.333
**GA/IAA**	32.582	<0.001	9.064	0.002	14.803	<0.001
**IAA/ABA**	17.049	<0.001	0.451	0.644	10.295	<0.001

Notes: *p* < 0.05 indicates a significant difference, *p* > 0.05 means no significant difference.

**Table 3:** Pearson correlation analysis of simulated salt stress on growth indicators, antioxidant system, and endogenous hormone ratio of *Wedelia trilobata* under different nutrient conditions.

	PH	LN	NN	RL	TB	RSR	Pro	MDA	POD	CAT	G/A	G/I	I/A
**PH**	1	0.737 **	0.755 **	0.368	0.767 **	−0.728 **	−0.419 *	−0.584 **	−0.288	−0.686 **	0.680 **	0.555 **	0.700 **
**LN**		1	0.658 **	0.007	0.517 **	−0.651 **	−0.288	−0.590 **	−0.150	−0.490 **	0.291	0.233	0.341
**NN**			1	0.316	0.694 **	−0.557 **	−0.414 *	−0.594 **	−0.144	−0.553 **	0.504 **	0.419 *	0.563 **
**RL**				1	0.610 **	−0.296	−0.355	−0.265	−0.460 *	−0.577 **	0.474 *	0.306	0.536 **
**TB**					1	−0.552 **	−0.494 **	−0.520 **	−0.653 **	−0.792 **	0.778 **	0.513 **	0.809 **
**RSR**						1	0.601 **	0.779 **	0.327	0.626 **	−0.501 **	−0.504 **	−0.532 **
**Pro**							1	0.684 **	0.275	0.374	−0.273	−0.480 *	−0.323
**MDA**								1	0.075	0.496 **	−0.387 *	−0.422 *	−0.467 *
**POD**									1	0.547 **	−0.450 *	−0.083	−0.489 **
**CAT**										1	−0.762 **	−0.374	−0.801 **
**G/A**											1	0.588 **	0.949 **
**G/I**												1	0.390 *
**I/A**													1

The PH: plant height; LN: leaf number; NN: number of nodes, RL: root length, TB: total biomass, RSR: root–shoot ratio, Pro: proline, MDA: malonaldehyde, POD: peroxidase; CAT: catalase; G/A: GA/ABA value; G/I: GA/IAA value; I/A: IAA/ABA value. The symbol of “*” indicates significant correlations (*p* < 0.05) and “**” signifies exceedingly momentous correlation (*p* < 0.01).

**Table 4:** Pearson correlation analysis of simulated salt stress on growth indicators, antioxidant system, and endogenous hormone ratio of *W. chinensis* under different nutrient conditions.

	PH	LN	NN	RL	TB	RSR	Pro	MDA	POD	CAT	G/A	G/I	I/A
**PH**	1	0.639 **	0.465 *	0.400 *	0.701 **	−0.417 *	−0.415 *	0.175	−0.507 **	−0.380	0.509 **	0.366	0.608 **
**LN**		1	0.694 **	0.362	0.674 **	−0.389 *	−0.232	0.183	−0.632 **	−0.310	0.501 **	0.123	0.652 **
**NN**			1	0.379	0.742 **	−0.354	−0.225	0.485 *	−0.800 **	−0.440 *	0.595 **	0.355	0.692 **
**RL**				1	0.672 **	−0.29	0.127	0.321	−0.684 **	−0.482 *	0.255	−0.134	0.476 *
**TB**					1	−0.293	−0.050	0.649 **	−0.831 **	−0.587 **	0.589 **	0.306	0.711 **
**RSR**						1	0.437 *	0.130	0.180	0.007	−0.258	−0.322	−0.293
**Pro**							1	0.141	0.097	−0.099	−0.384 *	−0.516 **	−0.259
**MDA**								1	−0.487 **	−0.268	0.301	0.192	0.281
**POD**									1	0.674 **	−0.702 **	−0.253	−0.829 **
**CAT**										1	−0.496 **	−0.187	−0.707 **
**G/A**											1	0.753 **	0.901 **
**G/I**												1	0.484 *
**I/A**													1

The PH: plant height; LN: leaf number; NN: number of nodes, RL: root length, TB: total biomass, RSR: root–shoot ratio, Pro: proline, MDA: malonaldehyde, POD: peroxidase; CAT: catalase; G/A: GA/ABA value; G/I: GA/IAA value; I/A: IAA/ABA value. The symbol of “*” indicates significant correlations (*p* < 0.05) and “**” signifies exceedingly momentous correlation (*p* < 0.01).

## Data Availability

Data are contained within the article.

## References

[1] Umair Hassan M., Chattha M.U., Khan I., Khan T.A., Nawaz M., Tang H., Noor M.A., Asseri T.A.Y., Hashem M., Guoqin H. (2024). Zinc seed priming alleviates salinity stress and enhances sorghum growth by regulating antioxidant activities, nutrient homeostasis, and osmolyte synthesis. Agronomy.

[2] Manjunath M.S., Koshariya A.K., Sharma N., Rajput A., Pandey S.K., Singh S., Kumar R., Singh B.V. (2023). Exploring the Use of Aromatic Compounds in Crop Growth and Protection. Int. J. Plant Soil Sci..

[3] Bouzroud S., Henkrar F., Fahr M., Smouni A. (2023). Salt stress responses and alleviation strategies in legumes: A review of the current knowledge. 3 Biotech.

[4] Verslues P.E., Bailey-Serres J., Brodersen C., Buckley T.N., Conti L., Christmann A., Dinneny J.R., Grill E., Hayes S., Heckman R.W. (2023). Burning questions for a warming and changing world: 15 unknowns in plant abiotic stress. Plant Cell.

[5] Kumari A., Ahlawat P., Rani B., Goyal A., Pazhany A.S., Kumar A., Devi S., Kumari N., Kiran, Pooja (2023). An Overview of Phytohormones Mediated Drought and Salinity Tolerance in Plants. Salinity and Drought Tolerance in Plants: Physiological Perspectives.

[6] Soto I., Balzani P., Carneiro L., Cuthbert R.N., Macedo R., Serhan Tarkan A., Ahmed D.A., Bang A., Bacela-Spychalska K., Bailey S.A. (2024). Taming the terminological tempest in invasion science. Biol. Rev..

[7] Yin W., Zhou L., Yang K., Fang J., Biere A., Callaway R.M., Wu M., Yu H., Shi Y., Ding J. (2023). Rapid evolutionary trade-offs between resistance to herbivory and tolerance to abiotic stress in an invasive plant. Ecol. Lett..

[8] Huang P., Hameed R., Abbas M., Balooch S., Alharthi B., Du Y., Abbas A., Younas A., Du D. (2023). Integrated omic techniques and their genomic features for invasive weeds. Funct. Integr. Genom..

[9] Pan L., He F., Liang Q., Bo Y., Lin X., Javed Q., Ullah M.S., Sun J. (2023). Allelopathic effects of caffeic acid and its derivatives on seed germination and growth competitiveness of native plants (*Lantana indica*) and invasive plants (*Solidago canadensis*). Agriculture.

[10] Shen C., Chen P., Zhang K., He M., Wan J., Wang Y., Tao Z., Huang W., Siemann E. (2023). Dynamics and mechanisms of secondary invasion following biological control of an invasive plant. New Phytol..

[11] Santos C.C., Basso Júnior I.J., Navarro V.L., Silva W.C., Silverio J.M., Scalon S.D.P.Q. (2023). Silicon Alleviates Damages on Photosynthetic Apparatus and Increases Resilience in Young *Inga vera* Plants Exposed to Water Deficit. J. Soil. Sci. Plant Nutr..

[12] Jianfan S., Qaiser J., Yizhou D., Ahmad A., Adeel A., Babar I., Yuhan H., Yan X., Daolin D. (2022). Invasive *Alternanthera philoxeroides* has performance advantages over natives under flooding with high amount of nitrogen. Aquat. Ecol..

[13] Mariyam S., Bhardwaj R., Khan N.A., Sahi S.V., Seth C.S. (2023). Review on nitric oxide at the forefront of rapid systemic signaling in mitigation of salinity stress in plants: Crosstalk with calcium and hydrogen peroxide. Plant Sci..

[14] Williams C.J. (2021). Phytochemistry of Australia’s Tropical Rainforest: Medicinal Potential of Ancient Plants.

[15] Chaudhry U.K., Gökçe Z.N.Ö., Gökçe A.F. (2021). Drought and salt stress effects on biochemical changes and gene expression of photosystem II and catalase genes in selected onion cultivars. Biologia.

[16] Ma J., Islam F., Ayyaz A., Fang R., Hannan F., Farooq M.A., Ali B., Huang Q., Sun R., Zhou W. (2022). Wood vinegar induces salinity tolerance by alleviating oxidative damages and protecting photosystem ii in rapeseed cultivars. Ind. Crop. Prod..

[17] Moraes G., De Almeida L.C. (2020). Nutrition and functional aspects of digestion in fish. Biology and Physiology of Freshwater Neotropical Fish.

[18] Zhang H., Goncalves P., Copeland E., Qi S., Dai Z., Li G., Wang C., Du D., Thomas T. (2020). Invasion by the weed conyza canadensis alters soil nutrient supply and shifts microbiota structure. Soil Biol. Biochem..

[19] Kaur H., Kaur H., Kaur H., Srivastava S. (2023). The beneficial roles of trace and ultratrace elements in plants. Plant Growth Regul..

[20] Jun S.E., Shim J.S., Park H.J. (2023). Beyond npk: Mineral nutrient-mediated modulation in orchestrating flowering time. Plants.

[21] Torres M., Parets S., Fernández-Díaz J., Beteta-Göbel R., Rodríguez-Lorca R., Román R., Lladó V., Rosselló C.A., Fernández-García P., Escribá P.V. (2021). Lipids in pathophysiology and development of the membrane lipid therapy: New bioactive lipids. Membranes.

[22] Song J., Fan Y., Li X., Li Y., Mao H., Zuo Z., Zou Z. (2022). Effects of daily light integral on tomato (*Solanum lycopersicon* L.) Grafting and quality in a controlled environment. Int. J. Agric. Biol. Eng..

[23] Qin H., Pandey B.K., Li Y., Huang G., Wang J., Quan R., Zhou J., Zhou Y., Miao Y., Zhang D. (2022). Orchestration of ethylene and gibberellin signals determines primary root elongation in rice. Plant Cell.

[24] Sharma B., Tiwari S., Kumawat K.C., Cardinale M. (2023). Nano-biofertilizers as bio-emerging strategies for sustainable agriculture development: Potentiality and their limitations. Sci. Total Environ..

[25] Kaniganti S., Bhattacharya J., Petla B.P., Reddy P.S. (2022). Strigolactone, a neglected plant hormone, with a great potential for crop improvement: Crosstalk with other plant hormones. Environ. Exp. Bot..

[26] Shahbani Z., Kosh-Khui M., Salehi H., Kafi M., Kamgar Haghighi A.A., Eshghi S., Omidi M. (2023). Hormonal and Physiological Changes in Miniature Roses (*Rosa chinensis* Jacq. var. minima Rehd.) Exposed to Water Deficit and Salinity Stress Conditions. Gesunde Pflanz..

[27] Khan I.U., Zhang Y., Shi X., Qi S., Zhang H., Du D., Gul F., Wang J., Naz M., Shah S.W.A. (2023). Dose dependent effect of nitrogen on the phyto extractability of Cd in metal contaminated soil using *Wedelia trilobata*. Ecotoxicol. Environ. Saf..

[28] Huang P., Wang H., Xu Z., Yang H., Du Y., Chen S., Abbas A., Yin H., Sun P., Du D. (2023). The responses of invasive *Wedelia trilobata* and native *Wedelia chinensis* to levofloxacin hydrochloride: Implication for biological invasion. Pol. J. Environ. Stud..

[29] Qi S., Manoharan B., Dhandapani V., Jegadeesan S., Rutherford S., Wan J.S.H., Huang P., Dai Z., Du D. (2022). Pathogen resistance in *Sphagneticola trilobata* (Singapore daisy): Molecular associations and differentially expressed genes in response to disease from a widespread fungus. Genetica.

[30] Sun S.W., Lin Y.C., Weng Y.M., Chen M.J. (2006). Efficiency improvements on ninhydrin method for amino acid quantification. J. Food Compos. Anal..

[31] Janero D.R. (1990). Malondialdehyde and thiobarbituric acid-reactivity as diagnostic indices of lipid peroxidation and peroxidative tissue injury. Free Radic. Biol. Med..

[32] Morales Hernandez C.E., Padilla Guerrero I.E., Gonzalez Hernandez G.A., Salazar Solis E., Torres Guzman J.C. (2010). Catalase overexpression reduces the germination time and increases the pathogenicity of the fungus *Metarhizium anisopliae*. Appl. Microbiol. Biotechnol..

[33] Konstantinou G.N. (2017). Enzyme-linked immunosorbent assay (ELISA). Food Allergens: Methods and Protocols.

[34] Singh A., Mehta S., Yadav S., Nagar G., Ghosh R., Roy A., Chakraborty A., Singh I.K. (2022). How to cope with the challenges of environmental stresses in the era of global climate change: An update on ROS stave off in plants. Int. J. Mol. Sci..

[35] Tyagi R., Pradhan S., Bhattacharjee A., Dubey S., Sharma S. (2022). Management of abiotic stresses by microbiome-based engineering of the rhizosphere. J. Appl. Microbiol..

[36] Ebert A.R., Frank D.A., Fridley J.D. (2023). Contrasting mycorrhizal growth responses in native and invasive woody species are associated with distinct root trait syndromes. Funct. Ecol..

[37] Huang P., He L., Abbas A., Hussain S., Hussain S., Du D., Hafeez M.-B., Balooch S., Zahra N., Ren X. (2021). Seed priming with sorghum water extract improves the performance of camelina (*Camelina sativa* (L.) Crantz.) Under Salt Stress. Plants.

[38] Sun J., Liu M., Tang K., Tang E., Cong J., Lu X., Liu Z., Feng Y. (2023). Advantages of growth and competitive ability of the invasive plant Solanum rostratum over two co-occurring natives and the effects of nitrogen levels and forms. Front. Plant Sci..

[39] Singh P., Kumar V., Sharma J., Saini S., Sharma P., Kumar S., Sinhmar Y., Kumar D., Sharma A. (2022). Silicon supplementation alleviates the salinity stress in wheat plants by enhancing the plant water status, photosynthetic pigments, proline content and antioxidant enzyme activities. Plants.

[40] Shen J., Wang Z., Su Y., Wang T. (2021). Associations between population epigenetic differentiation and environmental factors in the exotic weed mile-a-minute (*Mikania micrantha*). Weed Sci..

[41] Lekberg Y., Arnillas C.A., Borer E.T., Bullington L.S., Fierer N., Kennedy P.G., Leff J.W., Luis A.D., Seabloom E.W., Henning J.A. (2021). Nitrogen and phosphorus fertilization consistently favor pathogenic over mutualistic fungi in grassland soils. Nat. Commun..

[42] Guo Z., Miao W., Lyu Y., Wang X. (2024). Soil fungi lead to stronger ‘diminishing returns’ in fine-root length versus mass allometry towards earlier successional tropical forests. Funct. Ecol..

[43] Zhang A., Yin J., Zhang Y., Wang R., Zhou X., Guo H. (2023). Plants alter their aboveground and belowground biomass allocation and affect community-level resistance in response to snow cover change in Central Asia, Northwest China. Sci. Total Environ..

[44] Wells M.L., Karlson B., Wulff A., Kudela R., Trick C., Asnaghi V., Berdalet E., Cochlan W., Davidson K., De Rijcke M. (2020). Future HAB science: Directions and challenges in a changing climate. Harmful Algae.

[45] Young S.L., Anderson J.V., Baerson S.R., Bajsa-Hirschel J., Blumenthal D.M., Boyd C.S., Boyette C.D., Brennan E.B., Cantrell C.L., Chao W.S. (2023). Agricultural Research Service Weed Science Research: Past, Present, and Future. Weed Sci..

[46] Bowen J.L., Spivak A.C., Bernhard A.E., Fulweiler R.W., Giblin A.E. (2023). Salt marsh nitrogen cycling: Where land meets sea. Trends Microbiol..

[47] Bisht N., Mishra S.K., Chauhan P.S. (2020). *Bacillus amyloliquefaciens* inoculation alters physiology of rice (*Oryza sativa L. var*. IR-36) through modulating carbohydrate metabolism to mitigate stress induced by nutrient starvation. Int. J. Biol. Macromol..

[48] Wang Y., Yuan J., Li S., Hui L., Li Y., Chen K., Meng T., Yu C., Leng F., Ma J. (2021). Comparative analysis of carbon and nitrogen metabolism, antioxidant indexes, polysaccharides and lobetyolin changes of different tissues from *Codonopsis pilosula* co-inoculated with Trichoderma. J. Plant Physiol..

[49] Ahmad N., Naeem M., Ali H., Alabbosh K.F., Hussain H., Khan I., Siddiqui S.A., Khan A.A., Iqbal B. (2023). From challenges to solutions: The impact of melatonin on abiotic stress synergies in horticultural plants via redox regulation and epigenetic signaling. Sci. Hortic-Amst..

[50] Tomar R.S., Kataria S., Jajoo A. (2021). Behind the scene: Critical role of reactive oxygen species and reactive nitrogen species in salt stress tolerance. J. Agron. Crop Sci..

[51] Fatokun K., Beckett R.P., Varghese B., Cloete J., Pammenter N.W. (2020). Influence of cathodic water invigoration on the emergence and subsequent growth of controlled deteriorated pea and pumpkin seeds. Plants.

[52] Huang P., Xu Z., He W., Yang H., Li B., Ding W., Lei Y., Abbas A., Hameed R., Wang C. (2024). The Cooperation Regulation of Antioxidative System and Hormone Contents on Physiological Responses of *Wedelia trilobata* and *Wedelia chinensis* under Simulated Drought Environment. Plants.

[53] Hossain A., Pamanick B., Venugopalan V.K., Ibrahimova U., Rahman M.A., Siyal A.L., Maitra S., Chatterjee S., Aftab T. (2022). Emerging roles of plant growth regulators for plants adaptation to abiotic stress–induced oxidative stress. Emerging Plant Growth Regulators in Agriculture.

[54] Sahbeni G., Ngabire M., Musyimi P.K., Szekely B. (2023). Challenges and opportunities in remote sensing for soil salinization mapping and monitoring: A review. Remote Sens..

[55] Geng G., Li R., Stevanato P., Lv C., Lu Z., Yu L., Wang Y. (2020). Physiological and transcriptome analysis of sugar beet reveals different mechanisms of response to neutral salt and alkaline salt stresses. Front. Plant Sci..

[56] Hasan M.M., Gong L., Nie Z., Li F., Ahammed G.J., Fang X. (2021). ABA-induced stomatal movements in vascular plants during dehydration and rehydration. Environ. Exp. Bot..

